# Physicochemical characteristics and bronchial epithelial cell cytotoxicity of Folpan 80 WG^® ^and Myco 500^®^, two commercial forms of folpet

**DOI:** 10.1186/1743-8977-4-8

**Published:** 2007-09-20

**Authors:** Mireille Canal-Raffin, Beatrice L'Azou, Beatrice Martinez, Elisabeth Sellier, Fawaz Fawaz, Philip Robinson, Celine Ohayon-Courtès, Isabelle Baldi, Jean Cambar, Mathieu Molimard, Nicholas Moore, Patrick Brochard

**Affiliations:** 1 Laboratoire Santé-Travail-Environnement (EA 3672), Université Bordeaux 2, Bordeaux, France; 2 Département de Pharmacologie (INSERM U657), Université Bordeaux 2, Bordeaux, France; 3 Centre de Ressource en Microscopie Electronique et Microanalyse, Université Bordeaux 1, Bordeaux, France; 4 Laboratoire de Pharmacie Galénique et Biopharmacie (EA 3677), Université Bordeaux 2, Bordeaux, France

## Abstract

**Background:**

Pesticides, in particular folpet, have been found in rural and urban air in France in the past few years. Folpet is a contact fungicide and has been widely used for the past 50 years in vineyards in France. Slightly water-soluble and mostly present as particles in the environment, it has been measured at average concentration of 40.1 μg/m^3 ^during its spraying, 0.16–1.2 μg/m^3 ^in rural air and around 0.01 μg/m^3 ^in urban air, potentially exposing both the workers and the general population. However, no study on its penetration by inhalation and on its respiratory toxicity has been published. The objective of this study was to determine the physicochemical characteristics of folpet particles (morphology, granulometry, stability) in its commercial forms under their typical application conditions. Moreover, the cytotoxic effect of these particles and the generation of reactive oxygen species were assessed *in vitro *on respiratory cells.

**Results:**

Granulometry of two commercial forms of folpet (Folpan 80WG^® ^and Myco 500^®^) under their typical application conditions showed that the majority of the particles (>75%) had a size under 5 μm, and therefore could be inhaled by humans. These particles were relatively stable over time: more than 75% of folpet remained in the particle suspension after 30 days under the typical application conditions. The inhibitory concentration (IC_50_) on human bronchial epithelial cells (16HBE14o-) was found to be between 2.89 and 5.11 μg/cm^2 ^for folpet commercial products after 24 h of exposure. Folpet degradation products and vehicles of Folpan 80 WG^® ^did not show any cytotoxicity at tested concentrations. At non-cytotoxic and subtoxic concentrations, Folpan 80 WG^® ^was found to increase DCFH-DA fluorescence.

**Conclusion:**

These results show that the particles of commercial forms of folpet are relatively stable over time. Particles could be easily inhaled by humans, could reach the conducting airways and are cytotoxic to respiratory cells in vitro. Folpet particles may mediate its toxicity directly or indirectly through ROS-mediated alterations. These data constitute the first step towards the risk assessment of folpet particles by inhalation for human health. This work confirms the need for further studies on the effect of environmental pesticides on the respiratory system.

## Background

Pesticides are recognized as environmental pollutants, particularly in the ground and water [1]. The presence of pesticides has also been noted in the air by scientists and addressed by regulatory authorities [2,3]. However, in contrast with water, no obligatory monitoring or limitation of pesticide levels in air exists. Moreover, little is known about pesticide airway penetration and their impact on the respiratory system. Recently, public institutions developed pesticide air monitoring programs in several regions of France to characterize the level of exposure and to identify the principal compounds [4]. Theses studies collected PM_10 _(particulate matter collected with a 50% efficiency for particles with an aerodynamic diameter of 10 μm) and reported that in vine-growing regions during spring and summer (the treatment periods for vines) one of the main air-polluting pesticides was folpet [5-10]. In rural settlements, folpet was detected in the air at a large range of concentrations; mean levels were between 0.16–1.2 μg/m^3 ^depending on meteorological conditions, sprayed quantity and duration of treatment [5,8]. Folpet was also found in urban areas, the average concentration was around 0.01 μg/m^3 ^[6,7,9,10] excepted for the city of Reims (0.05–0.15 μg/m^3^) [5-8]. Such data indicates that the general population of these regions could be exposed. However, the most exposed populations are the workers who manipulate the product in the course of their work. The average concentration of folpet in the air during its spraying on crops was found to be 40.13 μg/m^3^(interquartile range: 1.7–14.95 μg/m^3^; maximum value: 857 μg/m^3^; Baldi, unpublished data).

**Figure 1:**
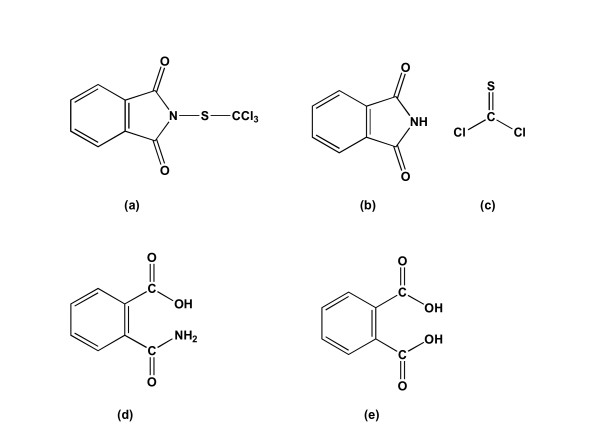
**Folpet and its degradation products adapted from Gordon [24]**. Folpet (a) is hydrolyzed to give (b) phthalimide and (c) thiophosgene. Phthalimide is then further hydrolyzed to give (d) phthalamic acid which is it-self hydrolyzed to give (e) phthalic acid.

Folpet (N-[(trichloromethyl)thio)phthalimide]) is a contact fungicide belonging to the dicarboximide family. It has been used for the past 50 years and is still widely employed in Europe as a preventive or curative treatment against mildew, gray mold, spoilage fungi and wood rot fungi [11]. Folpet is able to inhibit spore germination [12], its mode of action is centred on its reaction with thiol groups [13]. The folpet degradation pathway consists of hydrolysis with cleavage of the sulfur-nitrogen bond to give thiophosgene and phthalimide [14]. Phthalimide is hydrolyzed to phthalamic acid and then to phthalic acid (Fig. 1). Thiophosgene is a highly reactive short-lived compound, rapidly degraded to form HCl and SH_2 _in water.

Folpet is classified as a harmful substance, noxious by inhalation and with possible risks of irreversible effects [11,15]. Furthermore, studies performed *in vitro *on mammalian cells have shown folpet to induce cell-cycle deregulation [16], enzyme inhibition [17], clastogenic effects [18] and to have mutagenic effects)[19].

Nevertheless, many publications on folpet are relatively old and studies were not really performed using commercial forms of this fungicide. For agricultural treatment, folpet is generally available associated with one or two other fungicides and more rarely alone. It is formulated with vehicles as wettable powders, wettable granules (for example Folpan 80WG^®^) or suspension concentrates (for example Myco 500^®^) [11]. For use, these concentrated forms are diluted in water at a final concentration of 1 g/l and sprayed on vines or other crops such as apple trees [11]. At this manufacturer recommended working concentration, folpet is slightly water-soluble [13], remains on the surface of treated plants as particle form and acts as a contact fungicide. Depending on meteorological conditions and spray methods, air contamination can occur during application and, later on, by resuspension of folpet particles residing on treated areas. The general population can be exposed to folpet by this pathway, which might be particularly damaging to vulnerable persons.

The objective of this study was to determine the physicochemical characteristics of folpet particles of two commercial forms of folpet under their typical application conditions and to assess the cytotoxic effect *in vitro *on human cells. First, the granulometry and morphometry of the two commercial forms tested (Folpan 80WG^® ^and Myco 500^®^) were determined using analytical scanning electron microscopy and laser light diffraction methods. The stability of folpet particles and their degradation products were then evaluated using an analytical method specially developed for this study. These physicochemical characteristics led us to test the impact of these particles and/or degradation products on the respiratory tract using an *in vitro *model. Cultured human bronchial epithelial cells (16HBE14o-) were exposed to these particles; their cytotoxic effect was assessed using the neutral red release assay, the reactive oxygen species (ROS) detection was assessed using DCFH-DA stain.

## Results

### Morphometric analysis and particle size determination

**Figure 2:**
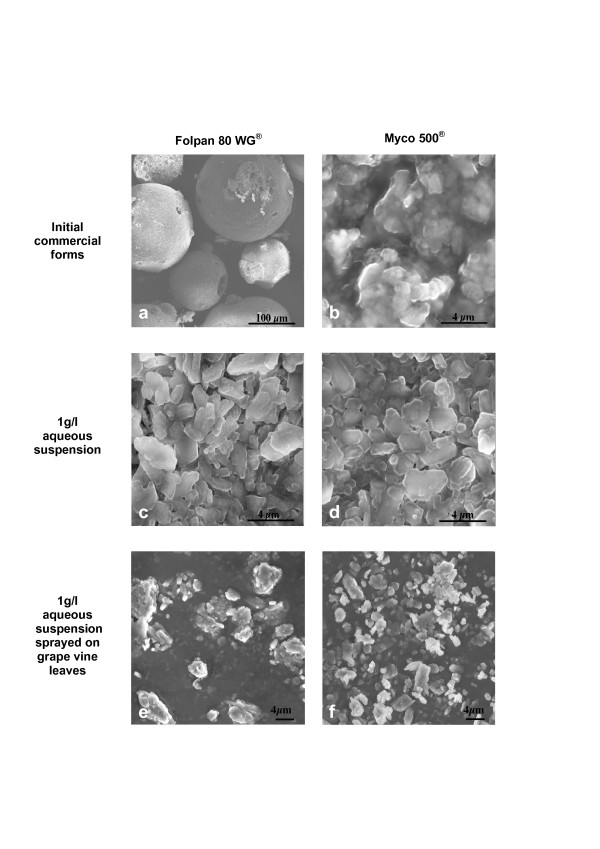
**Scanning-electron microscopy of folpet particles in two commercial forms at every step of their typical application conditions**. Both commercial forms of folpet are shown: (a, c and e) Folpan 80 WG^® ^and (b, d and f) Myco 500^®^; a and b respectively show Folpan 80 WG^® ^and Myco 500^® ^under their commercial forms; c and d show Folpan 80 WG^® ^and Myco 500^® ^under 1 g/l folpet aqueous suspension (manufacturer recommended dilution); e and f show Folpan 80 WG^® ^and Myco 500^® ^sprayed on grape vine leaves after 1 g/l aqueous suspension.

Scanning electron microscopy (SEM) observations of folpet particles were performed on two commercial fungicides available in France (Folpan 80WG^® ^and Myco 500^®^). Several observations for each product were performed under their commercial forms, under 1 g/l aqueous suspension and after spraying on grape vine leaves (Fig. 2).

Folpan 80 WG^® ^is a solid form of folpet, constituted by wettable granules which were found to be smooth and regular spherical particles without aggregation with a mean diameter of 233.7 ± 6.01 μm (Fig. 2a). More than 90% of folpet particles had a size between 100–500 μm. Myco 500^® ^is a liquid form of folpet constituted by a suspension concentrate of particles with a size of approximately 1–3 μm (Fig. 2b).

**Figure 3:**
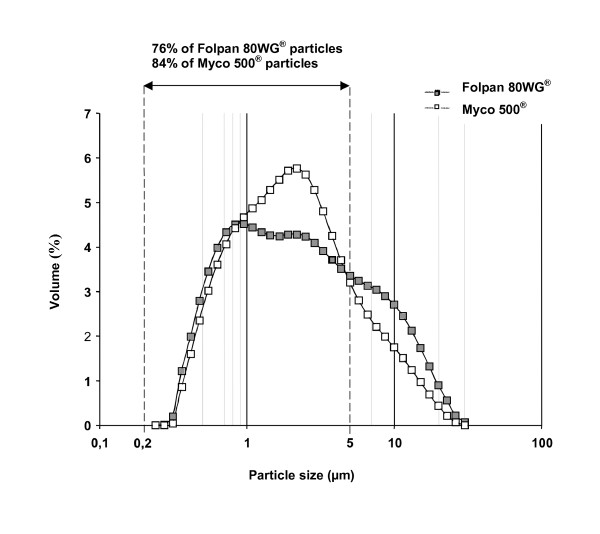
**Particles size distribution curve of Folpan 80WG^® ^and Myco 500^® ^in 1 g/l aqueous suspension**. The particle granulometry was measured by laser light diffraction and expressed by the volume mean diameter.

At the manufacturer recommended working concentration of 1 g/l aqueous suspension, folpet particles from the two commercial forms had similar morphometry (Fig. 2c, 2d). Size distribution of the two samples was determined by laser light diffraction: a monodisperse size distribution between 0.27–26 μm (Fig. 3), with a mean size of 3.70 for Folpan 80WG^® ^and 3.01 μm for Myco 500^® ^was observed. For Folpan 80WG^®^, 76% of the particles were smaller than 5 μm and for Myco 500^®^, this proportion was 84%.

**Figure 4:**
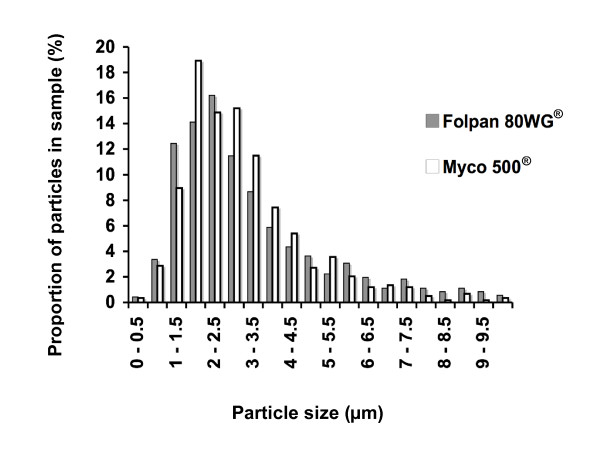
**Particle size distribution of Folpan 80WG^® ^and Myco 500^® ^sprayed on grape vine leaves**. Particles deposited on grape vine leaves were observed using a scanning electron microscope. Ten representative photographs of each sample were analyzed; the length of 715 particles from Folpan 80WG^® ^and 592 from Myco 500^® ^were measured using SIS Analysis^® ^software. The size distribution was not significantly different between Folpan 80WG^® ^and Myco 500^® ^(p = 0.1309, Wilcoxon test).

After spraying on grape vine leaves, particles from Folpan 80WG^® ^and Myco 500^® ^were observed by SEM. They had the same morphology (Fig. 2e and 2f) and a similar size distribution (p = 0.1309, Wilcoxon test) with a mean size of 3.6 μm (± 0.10) for Folpan 80WG^® ^and 3.0 μm (± 0.07) for Myco 500^® ^(Fig. 4). More than 80% of folpet particles were found to be under 5 μm in size for both commercial forms.

### Chemical stability of folpet particles and release of degradation products

**Figure 5:**
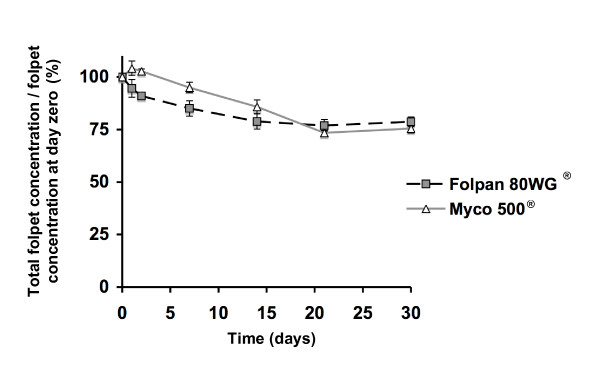
**Stability of folpet in aqueous suspension over time**. Total folpet concentration of Folpan 80WG^® ^and Myco 500^® ^suspended at 1 g/l of folpet in water (usual working concentration) was measured by HPLC-UV/DAD over 30 days and expressed as the proportion of the folpet concentration at day zero (%). Mean total folpet concentration (n = 5, ± se) is represented.

The chemical stability of folpet particles in Folpan 80WG^® ^and Myco 500^® ^was analysed under their typical application conditions: 1 g/l aqueous suspension at ambient temperature with a daylight/dark cycle over a period of 30 days (Fig. 5). At day zero there was no statistical difference (Student's t test, p = 0.6304) in total folpet concentration between Folpan 80WG^® ^(0.964 ± 0.03 g/l) and Myco 500^® ^(0.982 ± 0.02 g/l). At 30 days there remained 78.68% (± 2.32) of folpet in the Folpan 80WG^® ^sample and 75.47% (± 2.12) in the Myco 500^® ^sample, which was found not to be statistically different (Student's t test, p = 0.3374). Over the first 21 days, folpet degradation was more rapid than the days that followed and folpet in Folpan 80WG^® ^degraded more rapidly than in Myco 500^® ^(Fig. 5). The pH of the particle suspension started between 7.8 and 7.7 at day zero and decreased slowly to reach a value of 6.8 and 6.5 for Folpan 80WG^® ^and Myco 500^® ^respectively at day 30 (Table 2).

**Table 2:** pH of the particle suspension over time

**Days**	**0**	**2**	**7**	**14**	**21**	**30**
**Folpan 80 WG^®^**	7.8	7.4	7.1	6.9	6.8	6.8
**Myco 500^®^**	7.7	7.3	7	6.7	6.6	6.5

**Figure 6:**
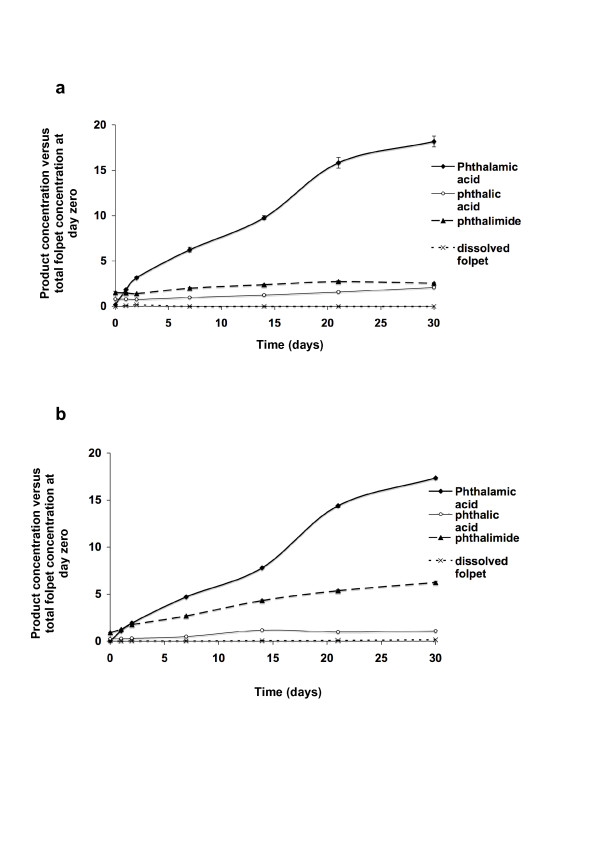
**Dissolved folpet and its released degradation products in water over time**. Dissolved folpet and its degradation products concentrations from (a) Folpan 80WG^® ^and (b) Myco 500^® ^suspended at 1 g/l in water were measured by HPLC-UV/DAD over 30 days. Product concentrations are expressed as a percentage of total folpet concentration at day zero (%). Mean concentrations (n = 5, ± se) are represented.

The concentration of folpet and the folpet degradation products in the dissolved fraction of Folpan 80WG^® ^and Myco 500^® ^suspensions were studied for 30 days (Fig. 6a, 6b). Dissolved folpet was detected at a very low level. The three known degradation products were detected at different concentrations. After 30 days, for both Folpan 80WG^® ^and Myco 500^®^, the degradation product found at the highest percentage of total folpet concentration at day zero was phthalamic acid (18.17% ± 0.602 and 17.36% ± 0.227, respectively) followed by phthalimide (2.55% ± 0.066 and 6.26% ± 0.102) and phthalic acid (2.09% ± 0.073 and 1.09% ± 0.060). At 30 days, there was no significant difference between Folpan 80WG^® ^and Myco 500^® ^with regard to phthalamic acid concentration (Student's t test, p = 0.2432), which was not the case for phthalimide and phthalic acid (Student's t test, p < 0.0001).

### In vitro studies

**Figure 7:**
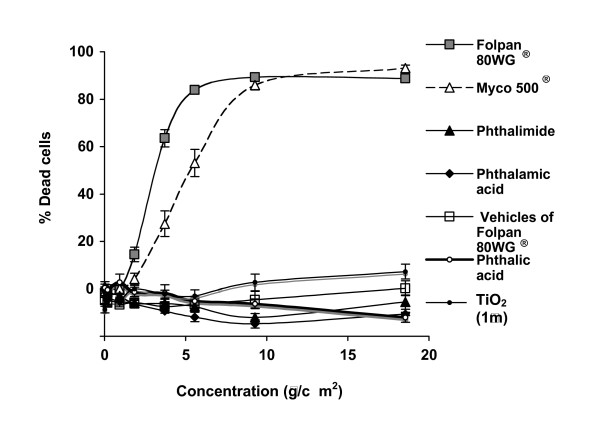
**Cytotoxic effect on 16HBE14o-cells after 24 h exposure**. The 16HBE14o-cells were exposed to Folpan 80WG^® ^(n = 31), vehicles of Folpan 80WG^® ^(n = 6), Myco 500^® ^(n = 15), phthalimide (n = 9), phthalamic acid (n = 9) and phthalic acid (n = 3) and micronic titanium dioxide (n = 3) during 24 h. A neutral red release assay was performed to quantify viable cells. The proportion of dead cells was calculated using the formula (100 - (Absorbance_540 nm–630 nm _drug-treated sample × 100/Absorbance_540 nm–630 nm _control sample)) and is expressed as mean ± se.

The 16-Human Bronchial Epithelial 14o-cells (16HBE14o-) were treated with various concentrations of folpet particles from Folpan 80WG^® ^and Myco 500^® ^for 24 h (Fig. 7). After 24 h exposure, folpet in its commercial forms induced cytotoxicity in a dose-dependent manner. The concentration of folpet in Folpan 80 WG^® ^and Myco 500^® ^required to induce 50% non viable cells compared with the control (IC_50_) was 2.89 μg/cm^2 ^(± 0.16) and 5.11 μg/cm^2 ^(± 0.36) respectively. The IC_50_between Folpan 80WG^® ^and Myco 500^® ^differed significantly (Student's t test, p < 0.0001). Vehicles of Folpan 80WG^® ^and the degradation products of folpet (phthalimide, phthalamic acid and phthalic acid) were tested separately under the same conditions. No cytotoxic effect was observed in the range of concentrations tested (0.185–18.5 μg/cm^2^) for both degradation products and vehicles of Folpan 80WG^® ^(Fig. 7). Micronic titanium dioxide used as negative control particles showed no cytotoxicity in the same range of folpet concentrations (0.185–18.5 μg/cm^2^).

**Figure 8:**
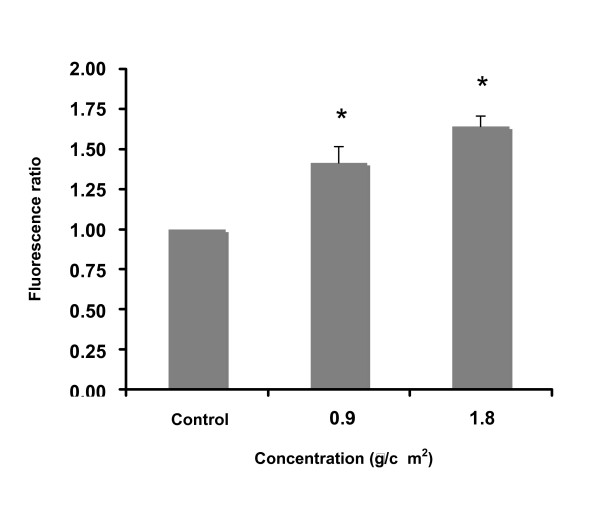
**ROS detection on 16HBE14o-cells as measured by DCFH-DA fluorescence levels after exposure to Folpan 80WG^® ^for 4 h**. Data are expressed as mean fluorescence ratios ± se (fluorescence of exposed cells/fluorescence of unexposed controls) for at least 3 independent experiments. * indicates statistical significance from unexposed control (<0.0001, ANOVA followed by a Dunnett's test).

Measuring DCFH-DA fluorescence, ROS were detected on 16HBE14o-cells following 4 h exposure to the most cytotoxic commercial form of folpet (Folpan 80WG^®^). At non toxic and subtoxic (IC_15_) concentrations, Folpan 80WG^® ^significantly increased DCFH-DA fluorescence to 1.41 ± 0.10 and 1.64 ± 0.06 fold of control fluorescence level respectively (p < 0.0001, one-way ANOVA followed by a Dunnett's post-test) (Fig. 8.). There was no statistically significant difference between the two tested concentrations (0.9 and 1.8 μg/cm^2^) for fluorescence increase (Newman-Keul post-test performed after one-way ANOVA).

## Discussion

Pesticides have only recently been considered as air pollutants in France [4] which has led folpet, a contact fungicide that has been widely used for the past 50 years, to be detected in rural and urban ambient air of several regions of France [20]. In light of this and in the absence of studies performed on commercial forms, we determined the physicochemical characteristics and cytotoxicity of two commercial forms of this fungicide (Folpan 80WG^® ^and Myco 500^®^). Here we report that these forms under their typical application conditions are composed of particles and that most of them are smaller than 5 μm, slightly water-soluble, stable over time and thus are potentially inhalable by the general population. Furthermore, these particles were found to be cytotoxic *in vitro *to human bronchial epithelial cells (IC_50 _= 2.9–5.1 μg/cm^2^) and to generate ROS production.

Testing of commercial forms of pesticides better resembles the real-life situation of their use. The two commercial forms of folpet tested here (Folpan 80WG^® ^and Myco 500^®^) were chosen because they contain only folpet as the active ingredient and because they differ in their initial formulations: the first is a wettable granulate (80% folpet w/w) and the second, a highly concentrated liquid suspension (500 g/l folpet). Studies performed on commercial forms of folpet and published in peer-review journals deal with the levels of residues on plants [21-23] not with the toxicity of these formulations on mammals. Yet several studies have been published using the technical grade molecule (for review see Gordon [24]). Here we report the first morphology and granulometry results of commercial forms of folpet fungicide formulations. The initial forms of Folpan 80WG^® ^and Myco 500^® ^differed morphologically. Yet once diluted in water to the manufacturer recommended working concentration of 1 g/l folpet and sprayed on grape vine leaves, both were found to be small polygonal particles, the majority of which less than 5 μm in size. Laser light diffraction, recognised as the most robust method for particles size determination was employed for the 1 g/l aqueous suspension. However, scanning electron microscopy was used for particles deposited on grape vine leaves after spraying owing to their solid state. The two methods gave similar granulometry results of either commercial form. These results were in close agreement with the measurement of folpet in air of French vine-growing regions as these studies collected PM_10 _[6-8].

We next tested the stability of these particles in an aqueous environment and found that even after 30 days, in a suspension of 1 g/l folpet of both Folpan 80WG^® ^and Myco 500^®^, more than 75% of the folpet remained. Folpet was found to be slightly water-soluble in accordance with previously published solubility data [13] and once dissolved, it degraded rapidly into different compounds. The loss of folpet in the total fraction was due to the dissolution of folpet particles in water and its subsequent hydrolysis. This was confirmed by a low level of folpet in the dissolved fraction and by an increased amount of degradation products over time. After 30 days, although the same quantity of folpet was degraded and all known degradation products were found [24], Folpan 80WG^® ^and Myco 500^® ^differed in their profile of degradation products. More phthalimide and less phthalic acid was found for Myco 500^® ^than for Folpan 80WG^®^. This may be explained by a more rapid folpet degradation in Folpan 80WG^® ^than in Myco 500^® ^during the first 21 days of our degradation study which itself may be due to a difference in vehicle composition. After 21 days, in the total fraction, there was no statistical difference in folpet concentration between Folpan 80WG^® ^and Myco 500^® ^and the rate of degradation was reduced to an inconsiderable level. This could be because the aqueous hydrolysis folpet is pH-dependent, the rate of folpet degradation is decreased at acidic pH [24,25]. The acidification found during our experiment could be a result of HCl released from the degradation of thiophosgene [26]. However, after 30 days the folpet in the dissolved fraction did not accumulate which could be due to an additional negative effect of the pH on the solubility folpet particles in water.

Taken together, we report here that the commercial forms of folpet, Folpan 80WG^® ^and Myco 500^®^, persist as particles in their typical application conditions and are stable in aqueous suspension. This could explain the presence of folpet (<1–50 ng/ml) in the aquatic environment [27] and in rural and urban air, during and after the period of treatment [28]. The risk of inhalation of particles by humans is determined by their size: particles under 10 μm are inhalable and those less than 5 μm are known to reach the deep lung and alveolar area but principally deposited on the bronchial area [29]. In their initial commercial forms, both Folpan 80WG^® ^and Myco 500^® ^could not be inhaled by humans because of the high size of Folpan 80WG^® ^granulates and the high concentrated suspension (500 g/l) with a doughy aspect for Myco 500^®^. However, at their recommended dilution and under their typical application conditions, 75% of the particles of both forms are less than 5 μm in size and could be inhaled.

It is suggested that folpet expresses its primary toxicity locally rather than by systemic effects [26]. However, few studies have been published on the cytological effects of folpet on mammalian cells [24]. Furthermore, to our knowledge, no study has specifically investigated the human respiratory system, which we addressed by using an *in vitro *model of human bronchial epithelial cells (16HBE14o-cells). These cells were chosen in accordance with the granulometric results for the particles.

The toxic effect of folpet was first investigated using an *in vitro *cytotoxicity test. This test allowed to study direct effects of particles on target cells following acute exposure. It is the starting point to provide mechanistic information and it is useful to define basal cytotoxicity giving the non-cytotoxic concentrations to be used for ROS detection. The estimated IC_50 _values for folpet on human bronchial epithelial cells (2.89 μg/cm^2 ^or 16 μM for Folpan 80WG^® ^and 5.11 μg/cm^2 ^or 30 μM Myco 500^®^) was similar to that reported for an other halogenated fungicide on human lung fibroblasts (chloropicrin, IC_50 _of 30 μM) [30]. Phthalimide, phthalamic acid and phthalic acid, the degradation products of folpet were found not to have a cytotoxic effect on 16HBE14o-cells, as well as the vehicles of Folpan 80WG^®^. These results support the hypothesis that thiophosgene contributed at least in part to the cytotoxic effect of folpet [31,32]. Further supporting this, we found that Folpan 80WG^® ^was more cytotoxic than Myco 500^® ^over 24 h which could be attributable to the differential kinetics of folpet degradation where more thiophosgene would have been released by Folpan 80WG^®^.

*In vivo *and *in vitro *studies have shown that the mechanism of toxicity of folpet could be due to a gluthation depletion [33] and enzyme alteration [17,34] by folpet reaction with thiol compounds [35]. In our study, folpet was found to generate ROS by increasing DCFH-DA fluorescence. This result supports the hypothesis that folpet may mediate its toxicity directly or indirectly through ROS-mediated alterations and cause an oxidative stress. This biological effect has been reported *in vivo *on an aquatic plants with a commercial form of folpet (Folpan 500^®^) [36]. This probable mechanism of toxicity has also been reported for several ambient air particulate matter pollutants like PM_2.5_or diesel exhaust particles (DEP) on respiratory cells [37-44]. Indeed, on 16HBE14o^- ^cells, from 10–30 μg/cm^2^, DEP and PM_2.5 _have been found to be potent inducers of oxidative stress with ROS production [42,44]. There is good evidence that these ambient PM can induce acute asthma exacerbation due to their ability to induce oxidative stress. ROS have been found to play an active role in the genesis of pulmonary inflammation and contribute to antigen-induced airway hyperreactivity [45,46]. Although concentrations of folpet particles in air are lower than those of PM_2.5_, folpet could have an additive effect or synergic effect with PM_2.5 _on oxydative stress production and then on respiratory toxicity. Moreover, folpet particles measured at concentrations 100-fold lower than PM_2.5 _in urban area (e.g. the city of Reims [47]) and only 10-fold lower in rural area, induce relatively high cytotoxicity on 16HBE14o-cells compared to other air pollutant particles. Indeed, from 10 to 30 μg/cm^2^, DEP and PM_2.5 _have been reported to have no effect on the viability of these cells [42,48] while, at these concentrations folpet particles have been found to induce more than 90% lethality. More studies should be performed to know whether the presence of folpet particles in air and particularly in rural area could represent a risk for the heath of this population.

The concentrations tested in our experiment were in the same range than other particles air pollutants like PM_2.5 _or DEP concentration (0.2–20 μg/cm^2^) tested on bronchial epithelial cells or macrophages [42,49]. Using calculations to relate *in vitro *DEP dose-response effects to *in vivo *PM dosimetry, Li and *al. *[49] reported that it is possible to achieve the *in vitro *dose range of 0.2–20 μg/cm^2^. When further corrections were made for the individual variations (e.g. deposition at airway bifurcation points, nasal breathing), PM_2.5 _were calculated to deposit in the tracheobronchial region at 2.3 μg/cm^2 ^for human subjects with uneven airflow (e.g. asthma) and at 1.13 μg/cm^2 ^for normal breathing individuals, exposed to an ambient total particulate matter average level of 79 μg/m^3^. Although, these calculations are based on 24 h exposure, workers exposed to an average concentration of folpet in air at 40.13 μg/m^3 ^during its manipulation could be probably exposed in the tracheobronchial region to folpet concentration closed to those inducing biological effects.

Using a personal air sampling pump, total suspended particles were collected to determine the potential inhalation exposure rate during folpet mixing and application. The time weighed average rate was evaluated to be 68.2 μg/h of work (IQR: 2.9–25.4 μg/h; maximum value: 1457 μg/h) during a working time average of 4.8 h (± 2.2) (Baldi, unpublished data). More research should focus on the workers, the most exposed population with an acute exposure to high concentration level during their work possibly in the range of concentration having biological effects.

On the other hand, the rural population in vine-growing regions less exposed than workers, but 10 to 100 fold more exposed than urban population should be monitored because little is known on the effect of a chronic exposure to these lower concentrations in humans. It is an important concern because the general population and particularly children could be exposed.

## Conclusion

The data presented here constitute the first step towards a risk assessment of folpet particles by inhalation for human health. Further studies are required, particularly to identify the mechanism of cytotoxicity and the *in vivo *impact on respiratory cells. Our data support the hypothesis that folpet found in the environment can persist, is inhalable by humans and is cytotoxic *in vitro *on human bronchial epithelial cells. The mechanism of toxicity of folpet could have been approached: folpet particles may mediate its toxicity directly or indirectly through ROS-mediated alterations. This work confirms the need for further studies on the effect of environmental pesticides on the respiratory system especially as no obligatory monitoring or limitation of pesticides in air exists as they do for water or food.

## Methods

### Tested products

Folpet, phthalamic acid, phthalic acid and titanium dioxide (1 μm) were obtained from Sigma-Aldrich (Saint Quentin-Fallavier, France), Phthalimide from Fluka (Saint Quentin-Fallavier, France) and were all analytical standards (>99% purity). Folpan 80WG^® ^(Makhteshim-Agan) and Myco 500^® ^(Capiscol), two commercial formulations containing respectively 80% (w/w) and 500 g/l folpet as the active ingredient were purchased from Euralis (Bruges, France). The vehicles of Folpan 80WG^® ^were kindly donated by Makhteshim-Agan.

### Morphometric analysis and particle size determination

The morphometric and granulometric determination of the particles were performed by two widely used methods [50,51].

#### Scanning electron microscopy

Morphometric analysis of folpet particles was performed at every step of the typical application conditions of the two commercial forms of folpet tested (Folpan 80WG^® ^and Myco 500^®^) before and after their suspension in water at 1 g/l (dose recommended by the manufacturer [11]), and after their spraying on grape vine leaves using a manual sprayer. For 1 g/l aqueous suspension, particles were gently vortexed for 1 minute and filtered using a 0.45 μm Nucleopore filter (Millipore, Saint Quentin-en-Yvelines, France). All samples were air-dried at room temperature before metal coating apart from Folpan 80WG^® ^which, in its commercial form, is a dry powder.

Samples of suspension concentrate Myco 500^® ^were deposited directly on the metal stub. Folpan 80WG^®^, nucleopore filters and parts of leaves were mounted onto the metal stub using double-faced adhesive tape. Samples were coated with a gold/palladium using a JFC-1100 ion-sputter (Jeol, Croissy-sur-Seine, France). Particle morphology was observed using a JSM-840A scanning electron microscopy (Jeol, Croissy-sur-Seine, France) operated at 10 KV. Ten representative photographs of each sample were analyzed. For Folpan 80WG^® ^and Myco 500^® ^sprayed on grape vine leaves, the length of more than 500 particles were measured using the SIS Analysis^® ^software (Olympus, Rungis, France).

#### Laser light diffraction

Before analysis, Folpan 80WG^® ^(25 mg) and Myco 500^® ^(40 μl) were independently suspended in 20 ml of ultra filtered water to yield a 1 g/l final folpet concentration and were gently vortexed for 1 minute. Folpet particle size distribution was measured by laser light diffraction (Mastersizer 2000, Malvern, England) and described by the volume mean diameter.

### Chemical stability studies

#### Reagents

Orthophosphoric acid and potassium dihydrogen phosphate were purchased from Merck (Fontenay Sous Bois, France), acetonitrile from JT Baker (Deventer, Holland) and methanol from Carlo Erba (Val de Reuil, France). All solvents were of high performance liquid chromatography (HPLC) grade. Deionised water was purified using a Milli-Q system (Millipore, Saint Quentin-en-Yvelines, France).

#### Sample preparations

In parallel, five 12 ml round-bottom polypropylene tubes containing 10 ml aqueous suspensions (1 g/l of folpet) of either Folpan 80WG^® ^or Myco 500^® ^were gently homogenized on a rotative agitator for 30 days, at ambient temperature with a daylight/dark cycle. Samples were taken immediately after preparation and at 1, 2, 7, 14, 21 and 30 days for the total folpet assay and for the dissolved compounds assay. The pH of each particle suspension was measured at the time of sampling using a pH meter (Hanna Instrument, Fisher Scientific Bioblock, Illkirch, France). For the total folpet assay, 10 μl of the particle suspension were vortexed in the presence of 500 μl acetonitrile to solubilise particles in order to quantify total folpet. To this, 490 μl of 10 mM KH_2_PO_4 _buffer (pH = 3.4, adjusted with H_3_PO_4_) were added to dilute and stabilize the sample before injection of 50 μl into the HPLC system.

For the dissolved compounds assay, 100 μl of the particle suspension were filtered using a GHP Nanosep^® ^MF (0.45 μm) filter unit (VWR, Fontenay Sous Bois, France) by centrifugation (10000 × g) for 1 minute. Ninety microlitres of the filtrate were then diluted with 100 μl methanol and 10 μl 10 mM H_3_PO_4_, 100 μl of this preparation were injected into the HPLC system.

The total folpet assay and the dissolved compounds assay were performed using the same analytical method.

#### HPLC-UV/DAD method

The HPLC system was an HP1100 model with a quaternary pump, an automatic injector and diode array ultra-violet detector (Hewlett Packard, Interchim, Montluçon, France), coupled with an HP ChemStation 6.0 system. Chromatographic analysis was performed at 25°C. The mobile phase consisted of an acetonitrile/10 mM KH_2_PO_4 _buffer (pH = 3.4, adjusted with H_3_PO_4_) gradient (Table 1) and was delivered at 1 ml/min flow rate. Before use, the mobile phase was filtered using a 0.2 μm nylon membrane. Compounds were separated on a dC18 Atlantis^® ^inversed phase column (150 × 4.6 mm, 5 μm, Waters, Saint Quentin-en-Yvelines, France) and detected at 218 nm for phthalimide, 224 nm for folpet and 200 nm for phthalic and phthalamic acid, within 19 min (retention time of phthalamic acid, phthalic acid, phthalimide, and folpet: 2.9, 4.3, 6.7 et 12.9 minutes, respectively).

Stock standard solutions of folpet and its degradation products were prepared at 1 mg/ml in acetonitrile for folpet, phthalimide, phthalic acid and in methanol for phthalamic acid. Working standard solutions were obtained by diluting stock standard solution under the same condition as the samples. Quantitative determinations were performed by measuring peak areas *versus *concentrations. This analytical method was specially developed and validated for this study. Good linearity was achieved in the range of 50–10000 ng/ml with correlation coefficients of 0.9976 (folpet, n = 3) in the total fraction, 0.9968 (phthalamic acid, n = 3), 0.9980 (phthalic acid, n = 3), 0.9972 (phthalimide, n = 3), 0.9985 (folpet, n = 3) in the dissolved fraction. Control samples were stored in a vial at room temperature on the auto injector HPLC system for 24 h. Folpet and phthalimide being unstable in vial, H_3_PO_4 _or 10 mM KH_2_PO_4 _buffer (pH = 3.4, adjusted with H_3_PO_4_) was added to the vial to prevent chemical degradation.

### In vitro studies

#### Cell culture conditions

The Human Bronchial Epithelial cells line sub clone 14o-(16HBE14o-) was kindly provided by Dr D. Gruenert. Cells were a SV40 large T antigen-transformed human bronchial epithelial cells, as described by Gruenert et al. [52]. These cells retained differentiated epithelial morphology and functions such as tight junctions, directional ion transport, a morphological polarity (microvillosity) and cytokeratine production but had lost cilia [53]. These cells are routinely employed to investigate the death and/or injury mechanisms on respiratory epithelial cells induced by environmental air contaminants [42,54-56]. Cells were maintained in EMEM (Eagle's Minimum Essential Medium, Cambrex, Verviers, Belgium) supplemented with 1% (v/v) penicillin (10^4^U/ml, Cambrex), 1% (v/v) streptomycin (10^4 ^μg/ml, Cambrex), 1% (v/v) fungizone (25 μg/ml, Cambrex), 1% (v/v) L-glutamine (200 mM, Cambrex) and 10% (v/v) heat-decomplemented foetal calf serum (FCS, Eurobio, Courtaboeuf, France) in a humidified atmosphere, 5% (v/v) CO_2_, at 37°C. To keep the cell morphological polarity, cells were seeded at 50 000 cells/cm^2 ^on 75cm^2 ^flasks (Greiner^®^) coated with 4 μg/cm^2 ^collagen (bovine type I, Becton Dickinson, Le Pont-de-Claix, France). Culture medium was replaced twice a week. Sub-confluent cells were released using trypsine/EDTA (500 mg/l/200 mg/l, Cambrex) during 10 minutes at 37°C.

#### Toxic exposures

Stock suspensions of Folpan 80WG^®^, vehicles of Folpan 80WG^®^, Myco 500^®^, micronic titanium dioxide, phthalimide, phthalamic acid or phthalic acid were prepared in serum-free culture medium. Cells were exposed to a range of 0.185–18.5 μg/cm^2 ^(active ingredient) concentrations corresponding to 0.1–100 μM, in serum-free culture medium, for 24 h. Concentrations were expressed in μg/cm^2 ^because particles rapidly deposed onto the cells.

#### Cell cytotoxicity test

Cytotoxicity was studied on sub-confluent cultures on collagen-coated 96 well plates (Falcon^®^) using the neutral red release assay according to Borenfreund et al. [57]. The neutral red release assay is an *in vitro *viablility test, based on the incorporation of neutral red strain into the lysosome of viable cells. This test is often used for assessing the cytotoxicity of contaminants such as pesticides [58] or particles [59] or gas [60].

After the exposure period, cells were washed with 200 μl/well of 0.9% (w/v) NaCl aqueous solution. M stock solution, prepared in 0.9% (w/v) NaCl aqueous solution, was filtered using a 0.22 μm filter in order to eliminate dye crystals. Neutral red stock solution was diluted 1:60 in serum-free EMEM and 200 μl were added to each well. After 3 h incubation, cells were rinsed with 0.5% (v/v) formaldehyde, 1% (w/v) CaCl_2 _aqueous solution. Cells were then lysed with 200 μl 1% (v/v) acetic acid, 50% (v/v) ethanol aqueous solution/well. Absorbance was measured at 540 nm (reference 630 nm) using a spectrophotometric microplate reader (Titertek multiskan^® ^plus, Labsystem, France).

#### Reactive oxygen specie (ROS) detection

Three days prior to each experiment, the 16HBE14o-cells were plated onto coated-plastic dishes 60 mm at 1.5 × 10^6 ^cells/plate to be 70–80% confluent at the start of the experiment. Cells were exposed to 0, 0.9 and 1.8 μg/cm^2 ^Folpan 80WG^® ^for 4 h in RPMI-1640 media without phenol red (Cambrex), 0% FCS, supplemented with 1% L-glutamine, 1% penicillin, 1% streptomycin and 1% fungizone in a humidified 5% CO_2 _incubator at 37°C.

ROS generation was measured by using 2',7'-dichlorodihydrofluorescein-diacetate (DCFH-DA, Sigma-Aldrich) as a probe. Before Folpan 80WG^® ^exposure, 1.2 ml of HBSS without phenol red, Ca^2+ ^and Mg^2+ ^(Cambrex), supplemented with 2 mM CaCl_2 _and 1 mM MgSO_4 _and containing 10 μM DCFH-DA was loaded for 15 min at 37°C.

DCFH-DA is a stable, non-fluorescent molecule that is hydrolyzed by intracellular esterases to non-fluorescent 2',7'-dichlorofluorescein (DCFH), which is rapidly oxidized in the presence of peroxides to a highly fluorescent adduct [61]. After 4 h Folpan 80WG^® ^exposure, the medium was collected and cells were scraped. Cells and culture medium were sonicated for 30 s. The fluorescence was measured in the supernatant using a spectrofluorimeter (SFM 25, Kontron instruments, Montigny le Bretonneux, France) with an excitation and emission wavelength of 480 and 520 nm, respectively. DCFH-DA results are reported as fluorescence ratio between exposed cells against unexposed cells.

### Statistical Analysis

All results are expressed as mean followed by standard error (mean ± se). Statistical analyses were performed with SAS 9.1 software (SAS Institute, North Carolina, USA).

For particle size distribution comparison of the two commercial forms of folpet, a non parametric test (Wilcoxon) was used. For the folpet stability study, normality test of Shapiro Wilk followed by a Student's t test were used for compounds concentration comparison.

For cytotoxicity experiments, neutral red release assay gave an absorbance signal (arbitrary unit; au) proportional to the number of viable cells within the well. All results were expressed as percentage of non-viable cells as calculated using this formula (100 - (Absorbance_540–630 nm _drug-treated sample × 100/Absorbance_540–630 nm _control sample)). The IC_50 _values (concentration required to induce 50% non viable cells compared with the control) were computed using the fitted equation with the Origin 6.0 software (Integral Sofware, Paris, France). A Shapiro Wilk normality test and a Student's t test were used for the IC_50 _comparison. One-way ANOVA followed by a Newman-Keuls post-test to perform multiple comparisons and a Dunnett's post-test to compare against non-exposed controls were used for ROS levels comparison.

## Competing interests

The author(s) declare that they have no competing interests.

## Authors' contributions

MCR carried out the SEM analysis, folpet particles stability study and *in vitro *cytotoxicity studies, analyzed the data and drafted the manuscript. BL assisted in *in vitro *cytotoxicity studies design and coordination. BM assisted in folpet particles stability study. ES helped to prepare all specimens for electron microscopy and perform SEM analyses. FF assisted in granulometric study. PR participated in the interpretation of results and drafting of the manuscript. OC, JC, IB, MM, NM participated in the interpretation of the results. PB assisted in study design, supervised the experimental work and coordination and helped in manuscript preparation. All authors read and approved the final manuscript.

**Table 1:** Mobile phase gradient of the HPLC-UV/DAD method

Time (min)	10 mM KH_2_PO_4 _buffer^a ^(%)	Acetonitrile (%)
0	**85**	**15**
0.5	**85**	**15**
9	**70**	**30**
11	**70**	**30**
13.5	**85**	**15**
17	**85**	**15**

^a ^pH = 3.4, adjusted with H_3_PO_4_
